# Human tumor infiltrating lymphocytes cooperatively regulate prostate tumor growth in a humanized mouse model

**DOI:** 10.1186/s40425-015-0056-2

**Published:** 2015-04-21

**Authors:** Michael D Roth, Airi Harui

**Affiliations:** Division of Pulmonary & Critical Care, Department of Medicine, David Geffen School of Medicine at UCLA, Los Angeles, CA 90095-1690 USA

**Keywords:** NOD/SCID/IL-2Rγnull, Human prostate cancer, Tumor infiltrating lymphocyte, Humanized

## Abstract

**Background:**

The complex interactions that occur between human tumors, tumor infiltrating lymphocytes (TIL) and the systemic immune system are likely to define critical factors in the host response to cancer. While conventional animal models have identified an array of potential anti-tumor therapies, mouse models often fail to translate into effective human treatments. Our goal is to establish a humanized tumor model as a more effective pre-clinical platform for understanding and manipulating TIL.

**Methods:**

The immune system in NOD/SCID/IL-2Rγnull (NSG) mice was reconstituted by the co-administration of human peripheral blood lymphocytes (PBL) or subsets (CD4+ or CD8+) and autologous human dendritic cells (DC), and animals simultaneously challenged by implanting human prostate cancer cells (PC3 line). Tumor growth was evaluated over time and the phenotype of recovered splenocytes and TIL characterized by flow cytometry and immunohistochemistry (IHC). Serum levels of circulating cytokines and chemokines were also assessed.

**Results:**

A tumor-bearing huPBL-NSG model was established in which human leukocytes reconstituted secondary lymphoid organs and promoted the accumulation of TIL. These TIL exhibited a unique phenotype when compared to splenocytes with a predominance of CD8+ T cells that exhibited increased expression of CD69, CD56, and an effector memory phenotype. TIL from huPBL-NSG animals closely matched the features of TIL recovered from primary human prostate cancers. Human cytokines were readily detectible in the serum and exhibited a different profile in animals implanted with PBL alone, tumor alone, and those reconstituted with both. Immune reconstitution slowed but could not eliminate tumor growth and this effect required the presence of CD4+ T cell help.

**Conclusions:**

Simultaneous implantation of human PBL, DC and tumor results in a huPBL-NSG model that recapitulates the development of human TIL and allows an assessment of tumor and immune system interaction that cannot be carried out in humans. Furthermore, the capacity to manipulate individual features and cell populations provides an opportunity for hypothesis testing and outcome monitoring in a humanized system that may be more relevant than conventional mouse models.

## Backgrounds

Tumor infiltrating lymphocytes (TIL) are a universal feature of human cancers and may hold an important key to unlocking effective cancer immunotherapy [1-3]. TIL exhibit tumor-specificity but are obviously impaired in their capacity to eradicate tumor targets *in situ* within the tumor microenvironment. While a basic understanding of the composition and phenotype of TIL has come from the study of human tumors, these studies are limited by patient heterogeneity, a lack of access to corresponding lymphoid tissue, and an inability to directly investigate mechanisms and interactions. As a result, researchers have turned to animal models to evaluate mechanisms and therapeutic outcomes [4-8]. These studies have provided important insights but even striking findings in animal models often fail to translate into useful clinically approaches. Xenograft models have allowed whole human tumor tissue, including TIL, tumor cells and other structural cells to be engrafted into immunodeficient mice and resulted in significant advances in understanding the human tumor microenvironment [9-14]. In one study [14], TIL from implanted tumor were able to migrate to spleen and maintained their characters even after adaptive transfer to another SCID mouse, providing a unique opportunity to investigate the function of TIL and test strategies to eradicate tumor.

The work presented in this study carries animal modeling one step further by simultaneously humanizing the immune system of recipient NOD/SCID/IL-2Rγnull (NSG) animals and challenging them with implanted human tumor cells. This humanized platform provides an opportunity to study the two-way interaction that occurs between human immunity and tumor growth, over time, and to manipulate individual components to test hypotheses and potential clinical impact. As a proof of concept, peripheral blood lymphocytes (PBL) and dendritic cells (DC) were obtained from healthy donors and used to reconstitute NSG animals, followed by implantation with cells from the human prostate cancer cell line, PC3. Resulting tumors demonstrated infiltration by TIL, with a composition and features very similar to those observed in tumor samples from prostate cancer patients, and reciprocal changes were observed in the spleens of tumor bearing animals suggesting both local and distant tumor responses. Serum from these animals contained cytokines produced by human lymphocytes as well as those by tumor, with further evidence of a two-way interaction. Finally, the rate of tumor growth was dependent upon both the presence and composition of the implanted lymphocytes.

## Results

### Human lymphocytes infiltrate and control tumor growth in huPBL-NSG animals

According to the experimental paradigm, animals were immune reconstituted with a combination of human PBL and DC alone, in combination with the subcutaneous (s.c.) implantation of PC3 cells, or with PC3 cells alone. PBL were prepared from peripheral blood mononuclear cells (PBMC) by depleting monocytes and natural killer cells (expressing CD14 and/or CD16) and activated T cells (CD25+) using monoclonal antibodies (mAbs) as described previously [15]. The resulting PBL consisted primarily of T cells (CD3+/CD56-; 72.7-90.2%), NKT cells (CD3+/CD56+; 2.8-9.8%), B cells (CD3-/CD20+; 3.5-8.0%), and a few NK cells (CD3-/CD56+; 1.0-2.2%). Monocyte-derived DC were CD14-negative and expressed high levels of class I and II major histocompatibility molecules and co-stimulatory molecules [15].

**Figure 1:**
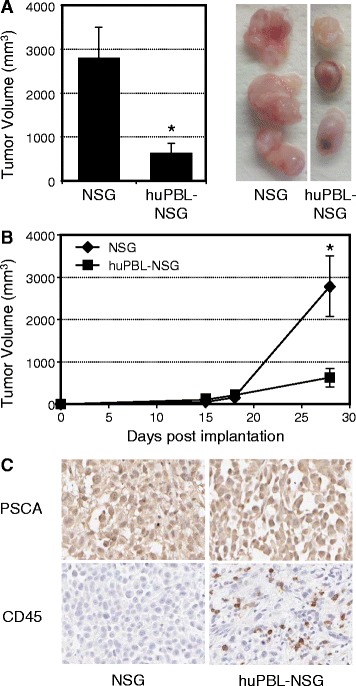
**Human PBL infiltrate and control tumor growth in huPBL-NSG animals.** Six-8-week- old NSG mice were injected with saline alone or with human PBL (1x10^7^) by intraperitoneal injection to produce huPBL-NSG animals. Mice were simultaneously implanted with 2x10^6^ human prostate tumor cells (PC3 cell line) by s.c. injection and tumor growth was monitored over a 4 week period. **(A)** Final tumor volume (mm^3^) as measured percutaneously in the animal and corresponding images of tumors dissected from NSG and huPBL-NSG animals at 4 weeks. **(B)** Tumor growth curves for NSG and huPBL-NSG animals. **(C)** Recovered tumors were formalin-fixed, paraffin-embedded,sectioned, and stained with antibodies against either human PSCA (top panel, brown stain), to demonstrate tumor cells, or human CD45 (bottom panel, brown stain), to demonstrate infiltrating human leukocytes, and counterstained with Hematoxylin (blue stain). Magnification, x100. Values represent mean of three animals per group ± SD. *p < 0.05. Representative experiment, n = 5 replicate experiments.

As shown in Figure 1A, the cross-sectional diameters of tumors recovered from huPBL-NSG mice were significantly smaller than tumors from NSG mice that were not immune reconstituted (average 620 ± 233 mm^3^ vs. 2792 ± 711 mm^3^; p < 0.05). When the rates of tumor growth over time were examined (Figure 1B), the difference between these two groups did not occur until at least two weeks after implantation, corresponding to the time at which functional immune reconstitution occurs in this model [15]. Immunohistochemistry (IHC) clearly demonstrated the presence of Prostate Stem Cell Antigen (PSCA) positive tumor in both cases and the presence of CD45+ human leukocytes infiltrating tumors harvested from huPBL-NSG animals (Figure 1C). As such, the presence of infiltrating human cells correlated with a reduction in the rate of tumor growth.

### TIL recovered from huPBL-NSG animals exhibit a unique phenotype distinct from splenocytes and similar to primary human TIL recovered from patients with prostate cancer

**Figure 2:**
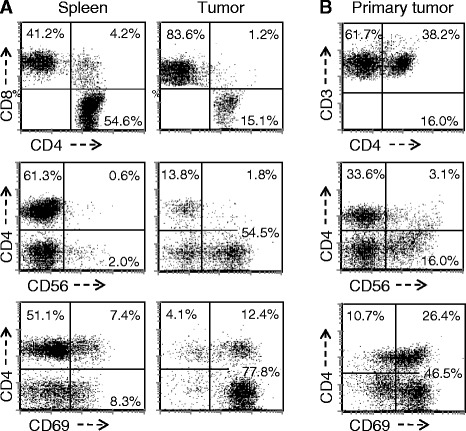
**Phenotype of human lymphocytes recovered from the spleen and tumor of tumor-bearing huPBL-NSG and from primary human prostate cancers.** Single cell suspensions were prepared from the spleen and tumor recovered from huPBL-NSG mice at 4 weeks **(A)** and from a primary human prostate cancer removed at the time of prostatectomy **(B)**. Cells were stained with an antibody cocktail, and then analyzed by flow cytometry for the expression of CD4 vs CD8 (top panel, gated on all cells expressing human CD4 and/or CD8); CD4 vs CD56 (middle panel, gate on all CD3+ cells); and CD4 vs CD69 (bottom panel, gated on all cells expressing human CD4 and/or CD8). The CD8+/CD56+ NKT population is identified by CD4-/CD56+ T cells. Representative experiment, n = 3 experiments.

The distribution and features of leukocytes that infiltrate human tumors are distinct from those present in peripheral blood or infiltrating normal tissues. Spleen cells and TIL were therefore recovered at 4 weeks after implantation and examined by flow cytometry. A representative example is presented in Figure 2A and documents the key features of TIL in this model. CD8+ T cells always predominated in TIL and the CD4:CD8 T cell ratio in TIL was always lower than that observed in spleen. While the splenic CD4:CD8 ratio was variable it ranged from 1.8-3.5 fold higher than that in the corresponding TIL (p < 0.005 by paired T test, n = 5 donors). In addition, TIL consistently exhibited a high percentage of cells exhibiting the CD8+/CD56+ NKT cell phenotype (34.02 ± 9.53%) while NKT cells represented only a small minority population within the spleen (5.76 ± 3.24%) and the difference was statistically significant (p < 0.00005, n = 6 experiments using different donors). In addition, the majority of TIL, regardless of whether they were CD4+, CD8+, or CD8+/CD56+ demonstrated the CD69 activation marker, while expression of this marker by splenic T cells was limited. B cells were rarely identified in TIL (<0.5%) nor were CD14+ monocytes (<0.3%), the later findings possibly reflecting the depletion of this population from the PBL used for implantation. While the cell populations and markers were distinctively different between tumor and spleen in tumor-bearing huPBL-NSG animals, the CD4/CD8 ratio and the presence of NKT cells did not differ between the spleens of control huPBL-NSG animals and those bearing PC3 tumors (data not shown). As such, TIL in this model exhibited a unique composition and features. In order to assess whether this recapitulated the features of native TIL we also performed FACS analysis on TIL obtained from patients with primary prostate cancer. As demonstrated in Figure 2B, TIL from prostate cancer also featured a CD8+ predominance with a high percentage of CD8 + CD56+ NKT and expression of the CD69 activation marker. Unfortunately, given the absence of matched spleen samples from patients, the features in human spleen from patients with prostate cancer cannot be directly commented on or compared – one of the important reasons for developing the humanized mouse model.

### The human cytokine and chemokine profile in huPBL-NSG animals is altered by the presence of tumor

**Figure 3:**
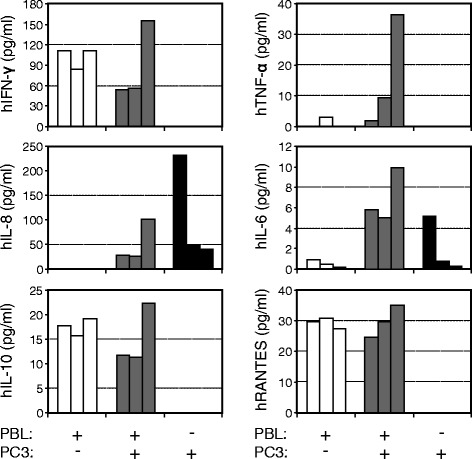
**Profile of human cytokines and chemokines in the serum of NSG and huPBL-NSG animals in the presence or absence of PC3 tumor implants.** Sera were collected from NSG animals 4 weeks after implantation of human PBL and/or PC3 tumor cells and assayed for the presence of human IFN-γ, TNF-α, IL-8, IL-6, IL-10 and RANTES by SearchLight multiplex assay. Representative experiment with each bar representing results from one animal, n = 3 experiments.

In contrast to the complexity of human serum, the human proteins present in the serum of huPBL-NSG animals can only originate from the cells that have been implanted and those expanded during the period of engraftment. As such, we hypothesized that serum measurements from these animals would directly reflect their immune reconstitution and its interaction with tumor (Figure 3). Sera were collected at 4 weeks from animals that were implanted with human PBL alone, those receiving both PBL and tumor, and animals that were implanted with tumor alone. hIFN-γ, hIL-10 and hRANTES were all present in huPBL-NSG animals and in huPBL-NSG animals that had been implanted with tumor, but not in animals that received PC3 cells alone. In contrast, while hTNF-α was detected at a low level in one of the huPBL-NSG animals it was detected in all of the huPBL-NSG animals that also had tumor implanted. This difference was not due to the tumor itself, which released no detectable hTNF-α when implanted alone, suggesting an immune cell-tumor interaction. The pro-inflammatory factor IL-8 appeared to be made primarily by the tumor and while IL-6 was released by both immune cells and tumor, its levels were more uniform and robust in reconstituted animals bearing PC3 tumors, again suggestive of an immune cell-tumor interaction. As the sample sizes were small and the distribution of cytokine levels were not always uniform, the mean values of each cytokine for each replicate animal are presented in Figure 3 in lieu of formal statistical analysis. These results suggest that the tumor-bearing huPBL-NSG model provides a sensitive platform for investigating the human cytokine and chemokine interactions relevant to the generation and maintenance of TIL.

### Human effector memory T lymphocytes (T-em) are increased in the TIL recovered from tumor-bearing huPBL-NSG animals

**Figure 4:**
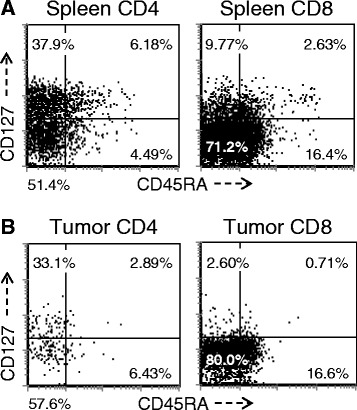
**T- naïve, T-cm and T-em subsets in the spleen and tumor.** Single cells were prepared from spleens **(A)** and tumors **(B)** recovered from huPBL-NSG mice 3 weeks after implantation and stained with antibodies against human CD3, CD4, CD8, CD45RA and CD127. Cells were gated for expression of CD3 and then analyzed by flow cytometry for the expression of other markers on the CD3+ population. Representative experiment, n = 5 experiments.

A more detailed analysis of T cell subsets was undertaken to evaluate for the presence of naïve (T-naïve, CD45RA^hi^/CD127^hi^), central memory T cells (T-cm, CD45RA^lo^/CD127^hi^) and T-em (CD45RA^lo^/CD127^lo^). As demonstrated in Figure 4, the majority of splenic CD4+ T cells in this model are split between T-cm and T-em, however there is always a recognizable T-naive population. In contrast, the T-em phenotype overwhelmingly predominates in TIL where the T-naive population was essentially absent and cells exhibiting a T-cm phenotype were limited. This overall pattern was similar for both the CD4+ and CD8+ T cells but most striking with respect to the CD8+ subset. While the difference between spleen and TIL subsets was numerically small, especially for the CD4+ population, it was highly reproducible and statistically significant when all mice that had been reconstituted with same PBL donor were compared (p < 0.01 for CD4+ T cells; p < 0.0001 for CD8+ T cells) and this pattern was significant in all experiments, with all donors when normalized by comparing the T-em: T-cm ratio (p <0.05 for both CD4+ T cells and CD8+ T cells). When primary tumors from patients were analyzed, the overwhelming majority of CD3+ T cells were CD45RA-negative (data not shown), which is consistent with the finding from our tumor-bearing huPBL-NSG model. In contrast, the majority of CD3+ T cells recovered from primary human tumors expressed CD127, more consistent with a T-cm rather than T-em phenotype. This likely reflects the sub-acute nature of the tumor-related interaction that occurs in this model, which is temporally different than the interaction between primary human tumors and infiltrating T cells *in vivo*, which occurs over a much longer period of time.

### Despite the predominance of CD8+ TIL, CD4+ T cells play an essential role in tumor regression in tumor-bearing huPBL-NSG animals

**Figure 5:**
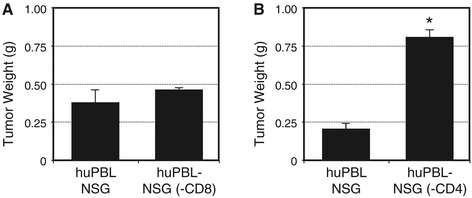
**Role of CD4+ and CD8+ lymphocyte subsets in controlling tumor growth.** NSG mice were reconstituted with either 1x10^7^ human PBL (huPBL-NSG) or PBL from the same donor that had been depleted of CD8+ T cells [huPBL-NSG (−CD8); **A]** or depleted of CD4+ T cells [huPBL-NSG (−CD4); **B]**. On the same day they received 2x10^6^ PC3 cells implanted by s.c. injection. Tumors were recovered after 3 weeks and individually weighed. Values represent mean of three animals per group ± SD. *p < 0.05. Representative experiment, n = 3 experiments.

**Figure 6:**
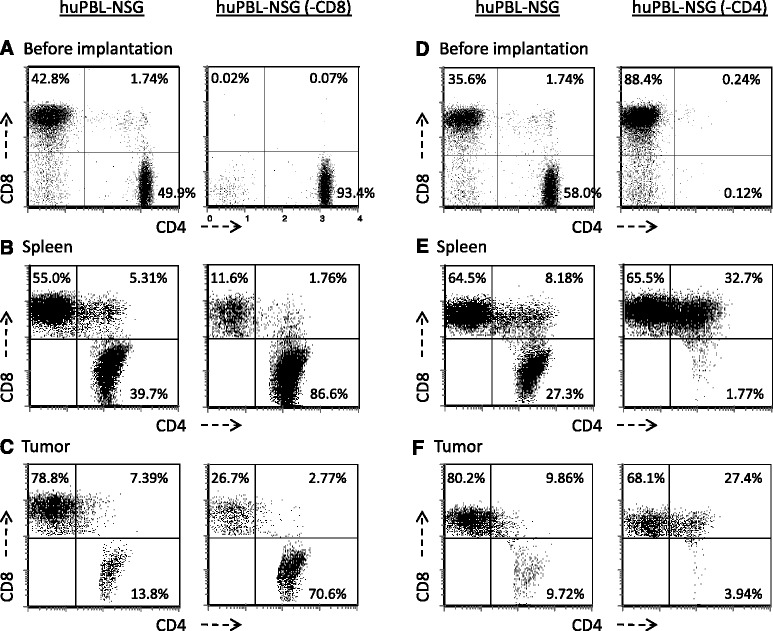
**Spleen reconstitution and tumor infiltration in NSG animals receiving purified human CD4+ or CD8+ T cells.** HuPBL-NSG, huPBL-NSG (−CD8) and huPBL-NSG (−CD4) animals were established as detailed in Figure 5 and implanted with 2x10^6^ PC3 cells by s.c. injection. **A & D**: The different purified PBL populations, before implantation, were stained with specific antibodies against human CD3, CD4 and CD8 followed by FACS analysis. Results are gated on the human CD3+ cells. Whole spleens **B & E** and tumors **C & F** were then recovered after 3 weeks and single cell suspensions stained with specific antibodies against human CD4 and CD8 followed by FACS analysis. Results were gated on all cells expressing CD4 and/or CD8. Representative experiment, n = 3 experiments.

Activated CD8+ T cells and NKT cells with a T-em phenotype predominate in TIL recovered from prostate tumors and in our tumor-bearing huPBL-NSG model. However, animal models have routinely suggested that CD4+ T cell help is essential for effective anti-tumor immunity. As a direct proof of principal, NSG mice were engrafted with either whole PBL or with PBL from the same donor that had been depleted of either CD8+ T cells [huPBL-NSG (−CD8)] or CD4+ T cells [huPBL-NSG (−CD4)]. As shown in Figure 5A, immune reconstitution with CD8-depleted PBL lead to the same control of tumor growth as did the administration of whole PBL. In addition, NSG animals that had been reconstituted with CD4-depleted PBL failed to control tumor growth (Figure 5B). Taken together, these results suggest that CD4+ T cells play an essential role in the immune response to tumor growth even though the majority of cells that accumulate within the tumor exhibit a CD8+ T-em phenotype. To better understand this phenomenon we examined the phenotype of T cells in the spleens and tumors of these animals (Figure 6). As shown in Figure 6A/B, even though CD8+ T cells were extensively depleted prior to implantation (<0.1% CD8+), there was a substantial population of CD8+ T cells (11-15% of total T cells) when cells were recovered from the spleens of reconstituted huPBL-NSG animals. Furthermore, as shown in Figure 6C there was still a preferential accumulation of CD8+ T cells in the tumor where the CD8+ population represented 25-35% of recovered T cells. As others have demonstrated in a variety of models [16,17], the repopulation of single-positive CD8+/CD4- T cells likely represents the rapid and marked homeostatic expansion of small numbers of contaminating CD8+ cells still present in the implanted PBL. On the other hand, the presence of double-positive CD4+/CD8+ T cells may reflect the *de novo* expression of CD8 that can occur when CD4+ cells are stimulated to expand [18]. As such, while CD4+ cells may be important we cannot rule out a significant contribution of the CD8+ T-em population with respect to controlling tumor growth. A related phenomenon appeared to occur in huPBL-NSG (−CD4) animals (Figure 6 D-F). While CD4+/CD8- cells did not regenerate when CD4+ cells were depleted prior to implantation, there was a striking increase of CD8+/CD4+ double-positive cells in spleen (32.7%) and tumor (27.4%) as compared to animals that received whole PBL where these double-positive cells only represented 8.2% and 9.9%, respectively. Moreover, unlike our consistent observation that the % of single CD8+ T cells was always greater in tumor than in spleen, there was no significant increase of CD8 T cells in tumor (68.1%) vs. spleen (65.5%) in huPBL-NSG (−CD4) mice, suggesting that the failure to expand tumor infiltrating CD8+ T cells contributed to the lapse in tumor control. Collectively, these results suggest a significant interaction between CD4+ and CD8+ T cell subsets is required with an essential role for both in effectively mediating anti-tumor responses.

## Discussion

TIL are present in all tumors and represent the endogenous immune response to cancer growth [1-3]. We hypothesized that huPBL-NSG animals might provide a unique opportunity to study the generation and function of human TIL *in vivo*. NSG mice lack high affinity receptors for IL-2, IL-4, IL-7, IL-9, IL-15 and IL-21 [19]. This deficit, when combined with their NOD/SCID background, provides an optimal environment for the engraftment of human cells including stem cells, PBL, DC and tumor cells [15,19,20]. Immune reconstitution with PBL is simple and reconstituted animals thrive without evidence of graft rejection or graft versus host disease when studied over a period of one to two months following implantation [15,19]. The combined administration of human PBL and DC, as in the current protocol, results in functional immune reconstitution including the capacity to generate and activate antigen-specific T and B cell responses [15,21]. Our first goal was to determine whether the engrafted human immune system and evolving tumor would interact in a manner that recapitulated the features of human TIL. Indeed, implanting human prostate cancer cells (PC-3 line) into huPBL-NSG animals resulted in a reproducible pattern of T cell trafficking, infiltration and activation that closely matched that observed in human prostate cancers. TIL recovered from tumors were characterized by a skewed CD4:CD8 T cell ratio with predominance of CD8+ T cells and CD8+/CD56+ NKT cells [22,23]. The expression of CD69, a marker of recent activation, was uniformly increased on recovered TIL but not by spleen cells, again recapitulating the pattern associated with human TIL *in vivo* [23,24]. However, while this model replicated many of the phenotypic features observed with primary human TIL, we observed the expression of a T-em phenotype in our model while TIL recovered from prostate cancer patients had a profile more consistent with T-cm cells. As others have reported a mixture of T-cm and T-em subsets in human TIL, this difference may relate to the sub-acute interaction that occurs in the model over several weeks rather than the chronic interaction of the tumor with infiltrating T cells that can occur over much longer time periods in patients with cancer [23]. Overall, the unique pattern of T cell infiltration and activation observed in TIL as compared to spleen suggests that the growing tumors and reconstituted immune cells are interacting in a manner that recapitulates the essential features of human TIL.

It is well established that TIL can have both prognostic value [25] and act as a target for cancer immunotherapy [26,27]. However, their contribution to the natural history of tumor growth is difficult to directly assess in patients. An important aspect of the humanized mouse model is the capacity to directly compare tumor growth in NSG animals, which lack immune function, with tumor growth in immune reconstituted huPBL-NSG animals generated from the same litter. While the T cell infiltration into the tumor was often subtle on IHC sections, their functional impact on tumor growth was striking. huPBL-NSG recipients were clearly protected from the rapid tumor expansion observed in tumor-bearing NSG controls. However, TIL failed to eradicate the tumor, suggesting a delicate balance between tumor-associated immune activation and tumor-associated immune suppression. TIL contain a variable mixture of activated, functionally tolerant and regulatory T cell and NKT cell subsets and the relative composition of these subsets is likely an important determinant of tumor growth [22,28-32].

In addition to phenotyping TIL and determining their impact on tumor growth, the huPBL-NSG model affords a unique opportunity to manipulate implanted immune cells and/or tumor to address mechanistic questions and test biologic hypotheses. As a proof of principal, we depleted CD8+ T cells from PBL prior to immune reconstitution. Implanted PBL contained only 0.03 to 0.09% CD8+ T cells as measured by flow cytometry (n = 5 donors). Animals reconstituted in this manner were still able to control tumor growth. Conversely, when CD4+ T cells were depleted, tumors grew at an accelerated rate similar to that observed in NSG animals that never received PBL. While these results might seem opposite of expectations, they are entirely consistent with the literature and with the type of immune reconstitution observed within their tumors and spleens. Using mouse models, investigators have described the essential collaboration that is required between CD4+ and CD8+ TIL subsets in order to mediate anti-tumor responses against prostate cancers [33]. While CD8+ T-em cells are the key effector population that mediate tumor killing, they can be skewed by the tumor environment to develop into regulatory and/or tolerant T cells in the absence of CD4+ T cell help. As such, when animals were reconstituted only with CD8+ cells there was limited control of the tumor even though we documented infiltration by CD8+ TIL. Interestingly, when animals were reconstituted with CD4-enriched T cells they still managed to engraft a substantial CD8+ population within the spleen and TIL. It is not clear whether these CD8+ T cells were derived from the hyper-expansion of trace numbers of CD8+ cells or whether other cell subsets were encouraged by the environment to acquire the CD8+ phenotype. However, in the presence of CD4+ helper cells, the level of CD8+ T cell reconstitution that occurs in these animals appeared sufficient to control tumor growth. A number of biological hypotheses regarding T cell subsets, function, and mediators can likely be assessed in this manner. Similarly, key features of the tumor responsible for T cell trafficking, immune activation and immune suppression might be assessed by implanting gene-modified tumor cells or through the administration of neutralizing antibodies directed against specific cytokines, chemokines or other regulatory pathways as suggested in related studies by Ye and coworkers [34].

NSG animals do not make human cytokines or chemokines and the presence of these factors in the serum of tumor-bearing huPBL-NSG animals must have derived from their implanted human tissues. Using serum collected at the end of each experiment we were able to reproducibly measure levels of hIFN-γ, hIL-10, hIL-6 and hRANTES in reconstituted huPBL-NSG animals. As expected, none of these cytokines were detected in NSG animals that were not immune reconstituted (data not shown). Cytokine profiles were entirely different when NSG animals were implanted with tumor alone (PC3-bearing NSG), where only tumor-derived IL-6 and IL-8 were detected in the serum. Of particular interest, hTNF-α appears primarily in the serum of tumor-bearing huPBL-NSG animals with limited detection in tumor-bearing NSG, suggesting that a TIL-tumor interaction is responsible for its production. Similarly, hIL-6 production appeared to increase in tumor-bearing huPBL-NSG animals when compared to levels in huPBL-NSG animals or tumor-bearing NSG. Human studies have reported that increased levels of hIL-6 and hTNF-α are found in prostate cancer patients with advanced tumors, especially in the setting of tumor-associated cachexia [35,36]. While our studies surveyed a range of cytokines in serum as a proof of concept, the tumor-bearing HuPBL-NSG model would also facilitate an analysis of local cytokine production within the tumor or spleen by extracting RNA or homogenizing samples for protein analysis.

## Conclusions

In summary, our findings suggest that implanting tumors into huPBL-NSG animals results in T cell trafficking, infiltration and activation within the tumor that occurs in a pattern mimicking that of native human TIL. The presence of TIL is associated with a marked slowing of tumor growth but does not result in tumor eradication, similar to the clinical pattern observed in patients with cancer. Manipulating the T cell subsets administered to these animals directly impacts on the capacity for their TIL to control tumor growth and the effects are consistent with our understanding of the role that different subsets play in tumor immunobiology. Serum monitoring in these animals detects the presence of human cytokines and factors secreted by their reconstituted immune cells, by the tumor, and the changes that occur due to their interaction. This model is simple, adaptable, and allows for highly controlled comparisons. Reconstitution of a mouse with 1x10^7^ PBL requires the equivalent of approximately 10 ml of donor blood and is usually complete within 2 weeks, simplifying the time and expense when compared to studies with purified cord blood stem cells which take months to engraft [19]. More importantly, this model provides access to the tumor, infiltrating cells, blood cells and spleen cells which can be used to investigate both local and systemic effects mediated by the interaction of cancer cells and host immunity. Based on the proof of concept testing reported here, the tumor-bearing huPBL-NSG model appears to have significant potential as a discovery platform for assessing the nature of human TIL and the interactions of TIL with the tumor that ultimately regulate tumor growth. However, even with all of these features this model has potential limitations. In the studies reported here we did not attempt to HLA match the tumor cell line and donor PBL and thus did not attempt to measure the generation of antigen-specific effector cells or identify antigen-specific T cell responses. This limitation can be overcome by HLA-matching of the PBL donor and the tumor, or by obtaining matched PBL and tumor cells directly from a cancer patient. In the latter setting, animals reconstituted with the blood and tumor of a patient would create a completely autologous xenograft model of the anti-tumor immunity that exists within the donor. Another limitation is that even though 10 million PBL are implanted, the reconstituted immune system cannot recapitulate the entire immune repertoire of the donor. However, as has been demonstrated with antigens such as influenza and adenovirus, there is every reason to belief that the model is fairly robust [15,21]. With extended time, a component of graft versus host disease will develop in huPBL-NSG animals [15] and studies should be limited to less than two months following PBL implantation. Alternatively, it has been proposed that NSG animals can be reconstituted with stem cells to produces a more stable, but naïve, immune reconstitution that can last the entire life of the animal [37,38]. However, such an approach still does not address the HLA mismatch with implanted tumor and in the one reported study the results appear very similar to those presented here [39]. A greater understanding of the interaction that occurs between human immunity and human tumors *in vivo* is needed and this model provides an interesting opportunity to rapidly advance our understanding of human tumor immunobiology.

## Methods

### Animals

Six- to 8-week-old NSG mice were bred and housed at UCLA under laminar flow conditions with all food, water (acidified), caging, and bedding autoclaved before use. No antibiotics were administered. All procedures were approved by the UCLA Animal Research Committee.

### Culture media and reagents

The human prostate cancer cell line, PC3 (kindly provided by Dr. Reiter, UCLA), was cultured with RPMI 1640 medium (Mediatech, Manassas, VA) supplemented with 10% FBS (Omega Scientific, Tarzana, CA), and penicillin–streptomycin–fungizone (Life Technologies, Carlsbad, CA). Human cells were cultured in RPMI 1640 supplemented with 10% human AB serum (Omega Scientific), penicillin–streptomycin–fungizone, and 10 mM HEPES (Life Technologies). Monoclonal antibody used for human T-and B-cell enrichment included anti-CD14, anti-CD16, and anti-CD25, and for DC enrichment included anti-CD3, anti-CD16, and anti-CD19, all from BD Biosciences (San Jose, CA). Anti-mouse Ig-conjugated immunomagnetic beads were from Life Technologies (M450 Dynabeads; Carlsbad, CA). Fluorochrome-conjugated mAbs against human CD3, CD4, CD8, CD56, CD69, and CD45RA were obtained from BD Biosciences; anti-CD127 from eBiosciences (San Diego, CA); and anti-CD25 from BioLegend (San Diego, CA,).

### Preparation of human lymphocytes and DC

Peripheral blood mononuclear cells were separated from the blood of healthy donors by density gradient centrifugation. Purified T-and B-cell subsets were further prepared by negative depletion using a mAb cocktail and anti-mouse Ig-conjugated immunomagnetic beads as previously described [15]. DC were prepared by culturing the adherent fraction of PBMC with 800 IU/ml granulocyte-macrophage colony stimulating factor (GM-CSF; Bayer Healthcare, Seattle, WA) and 63 ng/ml IL-4 (R&D Systems, Minneapolis, MN) for 5 days at 37°C as previously described [40]. Cells were recovered on day 5 and enriched by negative depletion using a mAb cocktail and anti-mouse Ig-conjugated immunomagnetic beads. Purified DC were cultured for an additional 48 hrs with GM-CSF and IL4 and activated during the final 24 hrs with the addition of human MegaCD40L (200 ng/ml, Alexis Biochemicals, Plymouth Meeting, PA) and interferon gamma (IFN-γ; 100 U/ml, PeproTech, Rocky Hills, NJ).

### Immune reconstitution and tumor implantation of NSG mice

NSG mice were implanted with 1x10^7^ human PBL (CD3+ or purified CD8+ or CD4+ T cells) in combination with 5×10^5^ autologous DC by intraperitoneal (i.p.) injection. PC3 prostate cancer cells (2x10^6^) were implanted on the same day into the right flank by subcutaneous (s.c.) injection.

### Analysis of human leukocyte subsets in the spleen and tumors of reconstituted NSG mice

Single-cell suspensions were prepared from the spleen and tumor by mechanical disaggregation. Cells were stained with fluorochrome-conjugated mAbs and results acquired with a modified FACScan flow cytometer (Becton-Dickinson, Franklin Lakes, NJ) with an added 637 nm diode and two associated detectors (Cytek Development Inc, Fremont, CA) and analyzed using FCS Express analysis software (DeNovo Software, Los Angeles, CA).

### In vivo release of human cytokines and chemokines

Serum samples were collected by retro orbital bleeding from animals at the time of sacrifice and assayed for the presence of human cytokines (IFN-γ, TNF-α, IL8, IL6, IL10) and a chemokine (RANTES) using a commercial SearchLight multiplex assay (Aushon Biosystems Inc, Billerica, MA).

### IHC staining of primary and implanted tumors

Fresh tumor specimens were obtained from patients undergoing prostatectomy through the UCLA Translational Pathology Core Laboratory in conjunction with an IRB-approved consent process. Tumors from NSG mice were recovered at the time of sacrifice, 3–4 weeks after implantation, fixed in 10% formalin and paraffin-embedded . IHC staining with specific antibody to human PSCA and CD45 was also carried out by the Translational Pathology Core Laboratory.

### Statistical analysis

Comparisons between groups were performed using Student’s t test, with p < 0.05 accepted as a significant difference between groups. All experiments were performed with replicates and results from at least three separate experiments were pooled for statistical analysis.

## Abbreviations

TIL: Tumor infiltrating lymphocytes
NSG: NOD/SCID/IL-2Rγnull
PBL: peripheral blood lymphocytes
DC: Dendritic cells
IHC: Immunohistochemistry
PSCA: Human Prostate Stem Cell Antigen
T-naïve: Naïve T cells
T-cm: Memory T cells
T-em: Effector memory cells
PBMC: Peripheral blood mononuclear cells
s.c.: subcutaneous
